# Beyond Cholesterol: Emerging Risk Factors in Atherosclerosis

**DOI:** 10.3390/jcm14072352

**Published:** 2025-03-29

**Authors:** Makhabbat Bekbossynova, Timur Saliev, Tatyana Ivanova-Razumova, Saltanat Andossova, Aknur Kali, Gulzhan Myrzakhmetova

**Affiliations:** 1 Heart Center, Corporate Fund University Medical Center, Nazarbayev University, Astana 010000, Kazakhstan; astanamaha@gmail.com (M.B.);; 2 Institute of Fundamental and Applied Medical Research, S.D. Asfendiyarov Kazakh National Medical University, Almaty 050000, Kazakhstan

**Keywords:** atherosclerosis, inflammation, gut microbiota, oxidative stress, environmental exposures, cardiovascular risk, lipid metabolism

## Abstract

Atherosclerosis remains a leading cause of cardiovascular morbidity and mortality worldwide, traditionally linked to elevated cholesterol levels, particularly low-density lipoprotein cholesterol (LDL-C). However, despite aggressive lipid-lowering strategies, residual cardiovascular risk persists, underscoring the need to explore additional contributing factors. This review examines emerging risk factors beyond cholesterol, including chronic inflammation, gut microbiota composition, oxidative stress, and environmental exposures. Inflammation plays a pivotal role in atherogenesis, with markers such as C-reactive protein (CRP), interleukin-6 (IL-6), and tumor necrosis factor-alpha (TNF-α) serving as indicators of disease activity. The gut microbiome, particularly metabolites like trimethylamine N-oxide (TMAO), has been implicated in vascular inflammation and plaque development, while beneficial short-chain fatty acids (SCFAs) demonstrate protective effects. Oxidative stress further exacerbates endothelial dysfunction and plaque instability, driven by reactive oxygen species (ROS) and lipid peroxidation. Additionally, environmental factors, including air pollution, heavy metal exposure, endocrine disruptors, and chronic psychological stress, have emerged as significant contributors to cardiovascular disease. Understanding these novel risk factors offers a broader perspective on atherosclerosis pathogenesis and provides new avenues for targeted prevention and therapeutic interventions.

## 1. Introduction

Among the many conditions that affect the cardiovascular system, atherosclerosis remains one of the most significant due to its association with coronary heart disease (CHD), myocardial infarction, and stroke [1]. Understanding the fundamentals of cardiology requires an appreciation of how the heart functions, the role of the vascular system, and the pathophysiology of diseases like atherosclerosis that compromise cardiovascular health. The heart is a muscular organ that pumps oxygenated blood to the body through the arterial system and returns deoxygenated blood to the lungs for oxygenation. The coronary arteries, which supply blood to the heart muscle itself, are particularly susceptible to atherosclerotic changes, making coronary artery disease (CAD) a leading cause of heart attacks [2]. The development of atherosclerosis involves a complex interplay between lipid metabolism, chronic inflammation, and endothelial dysfunction, ultimately leading to the formation of plaques within arterial walls.

At the molecular level, the accumulation of low-density lipoprotein cholesterol (LDL-C) within the arterial endothelium initiates an inflammatory cascade, attracting immune cells such as macrophages, which engulf LDL particles to form foam cells. Over time, these foam cells contribute to plaque formation, leading to arterial narrowing and impaired blood flow. When plaques rupture, they can trigger clot formation (thrombosis), resulting in acute cardiovascular events like myocardial infarction (heart attack) or ischemic stroke.

Beyond traditional cholesterol measures, lipoprotein(a) [Lp(a)] has emerged as an important biomarker for assessing cardiovascular risk. Unlike LDL-C, Lp(a) is genetically determined and has prothrombotic and pro-inflammatory properties, making it a significant contributor to atherosclerotic progression. While statin therapy effectively lowers LDL-C, Lp(a) remains largely resistant to standard lipid-lowering treatments, highlighting the need for novel therapeutic strategies.

Atherosclerosis is a progressive, chronic inflammatory disease of the arteries that remains a leading cause of coronary heart disease, cardiovascular morbidity, and mortality worldwide [3]. It is primarily characterized by the build-up of lipid-laden plaques within arterial walls, leading to vessel narrowing, reduced blood flow, and an increased risk of acute cardiovascular events such as myocardial infarction and stroke [4]. Historically, elevated cholesterol levels, particularly low-density lipoprotein cholesterol (LDL-C), have been considered the primary driver of atherosclerosis [5,6]. In addition, lipoprotein(a) [Lp(a)] has been considered a promising lipid marker beyond traditional cholesterol measures in assessing atherosclerosis risk [7].

Lp(a) is a genetically determined lipoprotein composed of an LDL-like particle and an apolipoprotein(a) [Apo(a)] component, which contributes to its pro-atherogenic, pro-inflammatory, and pro-thrombotic properties. Unlike LDL cholesterol, Lp(a) levels are largely unaffected by diet and lifestyle, making it a stable and independent predictor of cardiovascular disease (CVD) [8].

Elevated Lp(a) levels are strongly associated with accelerated atherosclerosis, coronary artery disease, stroke, and peripheral artery disease. Its structure allows it to promote plaque formation by increasing oxidized phospholipid content, fostering endothelial dysfunction, and enhancing foam cell formation. Additionally, Lp(a) interferes with fibrinolysis, contributing to thrombotic complications that further heighten CVD risk.

Traditional lipid assessments, including LDL-C and total cholesterol, often fail to fully capture residual cardiovascular risk, especially in patients with familial hypercholesterolemia or those with recurrent cardiovascular events despite statin therapy. Lp(a) measurement provides a valuable risk-stratification tool for these high-risk individuals, offering insights beyond standard lipid panels [7,9]. Currently, there are no widely approved pharmacological therapies specifically targeting Lp(a), though novel agents such as antisense oligonucleotides (ASOs) and small interfering RNAs (siRNAs) are in clinical development. These therapies aim to significantly reduce Lp(a) levels, potentially improving outcomes in high-risk CVD patients.

Consequently, much of the preventive and therapeutic focus has revolved around cholesterol management through lifestyle interventions and lipid-lowering medications such as statins. However, despite aggressive lipid-lowering strategies, a significant number of cardiovascular events still occur, underscoring the need to explore additional risk factors that contribute to the development and progression of atherosclerosis.

Emerging research suggests that atherosclerosis is a multifactorial disease influenced by various biological and environmental factors beyond cholesterol. Chronic inflammation, gut microbiota composition, oxidative stress, and environmental exposures have been identified as critical contributors to disease pathogenesis [10,11]. These factors interact in complex ways to influence endothelial function, immune responses, and lipid metabolism, ultimately shaping the trajectory of atherosclerosis. Understanding these novel contributors offers a more comprehensive perspective on cardiovascular disease risk and provides opportunities for targeted interventions that extend beyond conventional lipid management.

Among these emerging factors, inflammation plays a pivotal role in atherogenesis, with inflammatory markers such as C-reactive protein (CRP), interleukin-6 (IL-6), and tumor necrosis factor-alpha (TNF-α) serving as key indicators of disease activity [12,13]. Additionally, recent discoveries in gut microbiota research have highlighted the impact of microbial metabolites like trimethylamine N-oxide (TMAO) on cardiovascular health, suggesting that dysbiosis may be a modifiable risk factor for atherosclerosis [14,15]. Oxidative stress, driven by reactive oxygen species (ROS), exacerbates endothelial dysfunction and promotes plaque instability, further contributing to disease progression. Moreover, environmental exposures, including air pollution, heavy metals, endocrine disruptors, and chronic psychological stress have been increasingly recognized as external influences that accelerate vascular inflammation and plaque formation [16,17,18].

Given the multifaceted nature of atherosclerosis, it is essential to adopt a holistic approach that integrates these emerging risk factors into both clinical practice and public health strategies. Future research should focus on refining risk assessment models by incorporating inflammatory, microbial, oxidative, and environmental parameters, thereby improving cardiovascular disease prediction and prevention. This review aims to explore these novel risk factors in-depth, highlighting their mechanisms of action, clinical implications, and potential therapeutic targets. By broadening our understanding of atherosclerosis beyond cholesterol, we can develop more effective strategies for reducing the global burden of cardiovascular disease.

## 2. Inflammatory Markers and Atherosclerosis

Inflammation is now recognized as a fundamental driver of atherosclerosis, playing a pivotal role in every stage of the disease, from endothelial dysfunction to plaque rupture and thrombosis [19]. The vascular endothelium, a critical barrier between blood and tissues, responds to various insults, such as oxidative stress, hyperlipidemia, and infectious agents, by triggering an immune response that exacerbates plaque formation and progression. This chronic inflammatory process leads to persistent activation of immune cells, cytokine secretion, and arterial remodeling, which collectively contribute to atherosclerotic plaque development and instability [20,21].

Low-density lipoprotein cholesterol (LDL-C) is a well-established biomarker for cardiovascular risk, directly contributing to atherosclerotic plaque formation. However, LDL-C alone does not fully capture the complexity of atherosclerosis, as inflammation, endothelial dysfunction, and oxidative stress play critical roles in disease progression. Several biomarkers offer added value over LDL-C by providing insights into plaque instability, immune activation, and vascular damage, ultimately enhancing cardiovascular risk prediction and therapeutic strategies.

C-reactive protein (CRP) is a widely recognized marker of systemic inflammation, primarily produced by the liver in response to cytokine stimulation, particularly interleukin-6 (IL-6) (Table 1) [22]. Elevated CRP levels have been strongly associated with increased cardiovascular risk, even in individuals with normal lipid profiles. High-sensitivity CRP (hs-CRP) testing has emerged as a valuable tool for cardiovascular risk stratification, reinforcing the link between systemic inflammation and atherosclerosis [23]. The widespread use of high-sensitivity CRP (hs-CRP) testing for cardiovascular risk stratification could lead to many unnecessary tests and unwarranted interventions. Given that CRP levels can fluctuate due to transient infections or minor inflammatory processes, a single elevated hs-CRP reading may not accurately reflect long-term cardiovascular risk [24]. Consequently, this could result in excessive follow-up testing, additional imaging, and unnecessary prescriptions for anti-inflammatory or lipid-lowering therapies, increasing healthcare costs without clear benefits.

Furthermore, while some guidelines, such as those from the American Heart Association (AHA), suggest hs-CRP as a supplemental tool for cardiovascular risk assessment, others caution against its indiscriminate use due to its low specificity [25]. Population-level screening using CRP could lead to false positives, causing undue anxiety for patients and an overburdened healthcare system. More precise biomarkers or multi-marker approaches may be required to improve risk prediction and reduce unnecessary testing.

hs-CRP testing has emerged as a useful tool for risk stratification, particularly in individuals with normal lipid profiles but elevated inflammatory burdens. However, its predictive capacity is limited by non-specificity, as transient infections or minor inflammatory processes can cause fluctuations in CRP levels, leading to false positives and unnecessary testing. While some guidelines (e.g., AHA) recommend hs-CRP as a supplemental risk assessment tool, others caution against its overuse due to low specificity and potential overdiagnosis of cardiovascular risk.

IL-6 is a pro-inflammatory cytokine that plays a crucial role in amplifying vascular inflammation and endothelial dysfunction [26]. It promotes the production of acute-phase reactants such as CRP and fosters immune cell recruitment to atherosclerotic lesions. Studies have demonstrated that elevated IL-6 levels correlate with plaque vulnerability and adverse cardiovascular outcomes.

Tumor necrosis factor-alpha (TNF-α) is a potent mediator of endothelial activation, increasing vascular permeability and promoting the adhesion of leukocytes to the arterial wall. It induces the expression of adhesion molecules such as intercellular adhesion molecule-1 (ICAM-1) and vascular cell adhesion molecule-1 (VCAM-1), facilitating monocyte infiltration and plaque progression [27,28]. Moreover, TNF-α contributes to apoptosis and necrosis within atherosclerotic plaques, exacerbating their instability. Another important player is monocyte chemoattractant protein-1 (MCP-1), which plays a critical role in monocyte recruitment to sites of vascular injury [29,30]. Once monocytes enter the arterial intima, they differentiate into macrophages, ingest oxidized LDL, and form foam cells—a hallmark of atherosclerosis. Elevated MCP-1 levels have been linked to increased plaque burden and heightened cardiovascular risk. Additionally, interleukin-1 beta (IL-1β) is a key regulator of the inflammatory cascade in atherosclerosis, promoting endothelial dysfunction and smooth muscle cell proliferation [31]. The CANTOS (Canakinumab Anti-Inflammatory Thrombosis Outcomes Study) provided compelling evidence that targeting IL-1β with canakinumab, an IL-1β inhibitor, significantly reduced cardiovascular events independent of lipid levels, reinforcing the therapeutic potential of inflammation modulation [22,32].

Elevated IL-6 levels are associated with plaque vulnerability and adverse cardiovascular outcomes, making it a promising predictive marker for plaque instability and risk of acute coronary events. TNF-α plays a similar role in endothelial activation, increasing vascular permeability and enhancing immune cell adhesion to the arterial wall. Higher TNF-α levels have been linked to more aggressive atherosclerotic progression, suggesting its utility in predicting high-risk plaques and recurrent cardiovascular events. IL-1β acts as a central inflammatory mediator, promoting endothelial dysfunction and smooth muscle proliferation. The CANTOS demonstrated that targeting IL-1β with canakinumab significantly reduced cardiovascular events, confirming its role as a predictive and therapeutic target.

While traditional risk factors such as LDL cholesterol have been extensively studied, increasing evidence highlights the role of bioactive lipid mediators, particularly eicosanoids, leukotrienes, and thromboxanes, in amplifying the inflammatory processes underlying plaque formation and instability.

Leukotrienes, derived from arachidonic acid via the 5-lipoxygenase (5-LOX) enzyme pathway, are key mediators of vascular inflammation [33]. Leukotriene B4 (LTB4), a potent chemoattractant, facilitates monocyte and neutrophil recruitment to the endothelium, contributing to the inflammatory microenvironment that promotes foam cell formation and plaque development [34]. LTB4 also stimulates macrophages and dendritic cells, leading to increased secretion of pro-inflammatory cytokines such as tumor necrosis factor-alpha (TNF-α) and interleukin-6 (IL-6), which further exacerbate vascular damage [35,36]. In addition to LTB4, cysteinyl leukotrienes (CysLTs), including LTC4, LTD4, and LTE4, play a significant role in endothelial dysfunction by increasing vascular permeability, promoting smooth muscle cell proliferation, and inducing oxidative stress. These effects collectively contribute to the thickening of the arterial wall, loss of vascular elasticity, and eventual plaque rupture.

Beyond leukotrienes, eicosanoids such as thromboxanes also significantly influence atherosclerosis by modulating platelet activation and vascular tone. Thromboxane A2 (TXA2), synthesized via the cyclooxygenase (COX) pathway, is a potent vasoconstrictor and promoter of platelet aggregation [37]. Elevated levels of TXA2 have been associated with an increased risk of myocardial infarction and stroke, particularly in individuals with unstable plaques. The crosstalk between leukotrienes and thromboxanes further amplifies the inflammatory and thrombotic processes in atherosclerosis. LTB4 has been shown to enhance platelet activation, while TXA2 promotes leukocyte adhesion to the endothelium, creating a self-perpetuating cycle of inflammation, endothelial dysfunction, and thrombosis. Additionally, oxidized LDL (oxLDL), a critical driver of foam cell formation, has been found to stimulate 5-LOX activity, further increasing leukotriene production and promoting vascular inflammation [38,39]. Elevated levels of leukotrienes (LTs) and thromboxanes (TXs) have been linked to higher cardiovascular mortality, making them potential biomarkers for predicting plaque instability and thrombotic events.

Given the pivotal role of the leukotriene pathway in atherosclerosis, it presents a promising target for therapeutic intervention. Inhibitors of 5-LOX, such as zileuton, have been explored for their ability to suppress leukotriene synthesis, thereby reducing monocyte recruitment and inflammatory responses in the vasculature. Similarly, leukotriene receptor antagonists, including montelukast and pranlukast, commonly used in the treatment of asthma, have shown potential for mitigating vascular inflammation and endothelial dysfunction in preclinical models. Furthermore, dual inhibitors targeting both LOX and COX pathways offer a more comprehensive approach to reducing eicosanoid-mediated inflammation and thrombosis, potentially providing additional benefits in managing cardiovascular risk beyond conventional lipid-lowering therapies [40].

The leukotriene pathway represents a critical but underappreciated factor in atherosclerosis, influencing not only immune cell recruitment but also vascular remodeling and thrombotic risk. While current therapeutic strategies largely focus on reducing LDL cholesterol and controlling systemic inflammation with statins and anti-inflammatory agents, addressing specific lipid-derived inflammatory mediators such as leukotrienes and thromboxanes may offer new opportunities for reducing residual cardiovascular risk.

Given the established role of inflammation in atherosclerosis, novel therapeutic strategies aimed at dampening immune activation have gained considerable interest. Anti-inflammatory therapies, such as IL-1β inhibitors (e.g., canakinumab) and colchicine, an anti-inflammatory agent commonly used for gout, have shown promise in reducing cardiovascular risk [41]. Statins, in addition to lowering LDL-C, exert anti-inflammatory effects by reducing CRP levels and inhibiting immune cell activation. Lifestyle modifications, including regular physical activity, dietary interventions (e.g., a Mediterranean diet rich in anti-inflammatory polyphenols), and smoking cessation, can mitigate inflammation and improve vascular health. Additionally, emerging biologics and small molecules targeting specific cytokines and inflammatory pathways hold promise for future treatment strategies.

**Table 1:** Summary of the key inflammatory biomarkers in atherosclerosis, including their roles, advantages, and disadvantages.

Biomarker	Mechanism and Role	Clinical Relevance	Advantages	Disadvantages	Ref.
C-reactive protein (CRP)	Produced by the liver in response to IL-6; marker of systemic inflammation; associated with increased cardiovascular risk.	Used in risk stratification; high-sensitivity CRP (hs-CRP) is a strong predictor of cardiovascular events.	Widely available; non-invasive test; cost-effective.	Non-specific; elevated in various inflammatory and infectious conditions.	[23]
Interleukin-6 (IL-6)	Pro-inflammatory cytokine that amplifies vascular inflammation, promotes CRP production, and facilitates immune cell recruitment.	Correlates with plaque instability and adverse cardiovascular outcomes; potential therapeutic target.	Directly involved in inflammatory cascade; potential target for anti-inflammatory therapies.	Short half-life; highly variable levels; affected by multiple inflammatory conditions.	[22]
Tumor necrosis factor-alpha (TNF-α)	Enhances endothelial activation, increases vascular permeability, promotes leukocyte adhesion, and contributes to plaque formation and rupture.	Elevated TNF-α levels are associated with increased atherosclerosis severity and cardiovascular mortality.	Well-established role in inflammation; TNF-α inhibitors exist and are widely used in autoimmune diseases.	Systemic effects can lead to immunosuppression; TNF-α inhibitors can have severe side effects such as infections and malignancies.	[27]
Monocyte chemoattractant protein-1 (MCP-1)	Key player in monocyte recruitment to atherosclerotic plaques, promoting foam cell formation and chronic inflammation.	Elevated MCP-1 levels correlate with increased plaque burden and cardiovascular events.	Plays a crucial role in early-stage atherogenesis; potential biomarker for identifying high-risk individuals.	Not widely used in routine clinical practice; limited availability of standardized assays.	[29,30]
Interleukin-1 beta (IL-1β)	Central mediator of inflammation; activates endothelial cells and smooth muscle proliferation, promoting plaque growth and instability.	Targeted in the CANTOS, where IL-1β inhibition (canakinumab) significantly reduced cardiovascular events.	Strong potential for therapeutic targeting; IL-1β inhibitors have shown clinical benefits beyond lipid lowering.	Expensive treatment; inhibition can weaken immune defenses, increasing infection risk.	[31]
Intercellular adhesion molecule-1 (ICAM-1)	Facilitates leukocyte adhesion to the endothelium, aiding immune cell infiltration into plaques.	High ICAM-1 levels are linked to increased endothelial dysfunction and cardiovascular risk.	Useful in studying vascular inflammation; potential marker for endothelial activation.	Lacks specificity for atherosclerosis; limited role in routine clinical settings.	[42]
Vascular cell adhesion molecule-1 (VCAM-1)	Promotes monocyte and T-cell adhesion to endothelial cells, accelerating plaque development.	Higher VCAM-1 levels correlate with early atherosclerosis and plaque progression.	Strongly associated with vascular inflammation; may help identify subclinical disease.	Not routinely measured in clinical practice; affected by various inflammatory conditions.	[43]
Myeloperoxidase (MPO)	Enzyme released by neutrophils that promotes oxidative stress, endothelial dysfunction, and LDL oxidation.	Elevated MPO levels predict future cardiovascular events and plaque vulnerability.	Provides insights into oxidative stress-driven inflammation; could serve as a marker for plaque instability.	Less commonly used; testing is not standardized in routine cardiovascular screening.	[44]
Lipopolysaccharide-binding protein (LBP)	Indicator of bacterial endotoxin activity; linked to gut microbiota dysbiosis and systemic inflammation.	Higher LBP levels are associated with metabolic syndrome, obesity, and increased atherosclerotic risk.	Highlights gut-immune interactions in atherosclerosis; potential target for novel therapies.	Research is still emerging; clinical applications are limited.	[45]

Several clinical trials have investigated the role of inflammatory biomarkers in cardiovascular disease, particularly in relation to atherosclerosis, risk prediction, and targeted anti-inflammatory therapies. These studies have provided valuable insights into the clinical relevance of inflammatory markers such as C-reactive protein (CRP), interleukin-6 (IL-6), tumor necrosis factor-alpha (TNF-α), monocyte chemoattractant protein-1 (MCP-1), interleukin-1 beta (IL-1β), leukotrienes, and thromboxanes. While some trials have explored their predictive capacity for cardiovascular events, others have assessed the efficacy of therapies aimed at reducing inflammation to improve cardiovascular outcomes.

One of the most well-known trials investigating inflammatory biomarkers in cardiovascular disease is the above-mentioned CANTOS (Canakinumab Anti-Inflammatory Thrombosis Outcomes Study) [46,47]. This landmark study evaluated the effects of IL-1β inhibition using canakinumab in patients with a history of myocardial infarction and elevated high-sensitivity CRP (hs-CRP) levels.

The JUPITER (Justification for the Use of Statins in Prevention: an Intervention Trial Evaluating Rosuvastatin) also provided significant evidence supporting the role of inflammation in cardiovascular disease [48]. This study investigated whether rosuvastatin could reduce cardiovascular events in individuals with normal LDL-C levels but elevated hs-CRP. The results showed that patients who received statin therapy experienced a significant reduction in cardiovascular risk, suggesting that hs-CRP could serve as a useful biomarker for identifying high-risk individuals who might benefit from lipid-lowering and anti-inflammatory treatment, even in the absence of hyperlipidemia.

The COLCOT (Colchicine Cardiovascular Outcomes Trial) evaluated colchicine, an anti-inflammatory agent commonly used for gout, in patients with recent myocardial infarction [49,50]. The trial demonstrated that low-dose colchicine significantly reduced the risk of major cardiovascular events, further confirming that inflammation plays a crucial role in atherosclerosis progression. Colchicine’s inhibition of IL-1β, IL-6, and other inflammatory pathways provided strong evidence that targeting inflammation, rather than just lowering cholesterol, could improve cardiovascular outcomes.

Another important study is the LoDoCo2 (Low-Dose Colchicine for Secondary Prevention of Cardiovascular Disease) trial, which examined colchicine’s long-term effects on stable coronary artery disease [51,52]. The results showed a 30% reduction in cardiovascular events, reinforcing the potential of chronic anti-inflammatory therapy as a viable strategy for secondary prevention in patients with established atherosclerosis.

Beyond CRP, IL-6, and IL-1β, clinical trials have also explored other inflammatory mediators such as leukotrienes and thromboxanes. Leukotriene-modifying agents, such as zileuton (a 5-lipoxygenase inhibitor) and montelukast (a leukotriene receptor antagonist), have primarily been used in asthma treatment, but emerging research suggests their potential role in reducing vascular inflammation [53,54]. Some early-stage studies have investigated whether leukotriene inhibitors could reduce plaque progression or improve endothelial function, though large-scale cardiovascular trials are still needed.

Additionally, trials targeting thromboxane A2 (TXA2), a key mediator of platelet aggregation and vascular constriction, have explored whether thromboxane inhibitors could reduce cardiovascular events [55]. Aspirin, which inhibits thromboxane synthesis via cyclooxygenase (COX) inhibition, has long been a cornerstone of cardiovascular disease prevention. However, more selective thromboxane receptor antagonists, such as terutroban, have been investigated for their ability to further reduce thrombotic risk, though their clinical benefits remain uncertain compared to aspirin.

Ongoing and future trials continue to investigate the potential of multi-marker approaches that combine traditional lipid profiles with inflammatory biomarkers to enhance cardiovascular risk prediction. Researchers are also exploring the integration of genetic and proteomic data to refine risk assessment and develop personalized anti-inflammatory treatment strategies.

While LDL-C remains a cornerstone of cardiovascular risk assessment, incorporating inflammatory, endothelial, and oxidative stress biomarkers can enhance risk prediction, allowing for more personalized prevention and treatment strategies. A multi-biomarker approach that integrates LDL-C with markers such as CRP, IL-6, TNF-α, ICAM-1, and MPO may provide a more comprehensive assessment of cardiovascular risk, optimizing clinical decision making and improving patient outcomes.

In fact, while LDL-C quantifies lipid burden, CRP identifies ongoing vascular inflammation, making it particularly valuable for assessing risk in individuals with low to moderate LDL levels. Interleukin-6 (IL-6) is a key cytokine driving vascular inflammation and endothelial dysfunction, serving as a more direct indicator of inflammatory activity than LDL-C alone. TNF-α further amplifies inflammation by increasing endothelial permeability and immune cell adhesion, contributing to plaque progression and instability.

Monocyte chemoattractant protein-1 (MCP-1) is central to immune cell recruitment in atherosclerosis, as it facilitates monocyte infiltration into plaques. Unlike LDL-C, which measures lipid accumulation, MCP-1 reflects the inflammatory microenvironment within plaques, making it useful for predicting early-stage atherosclerosis. Similarly, interleukin-1 beta (IL-1β) plays a crucial role in chronic vascular inflammation, with studies like the CANTOS demonstrating that IL-1β inhibition can significantly reduce cardiovascular events. This suggests that targeting inflammation may be as important as lowering LDL-C in preventing disease progression.

Endothelial dysfunction is a major contributor to atherosclerosis, and biomarkers such as intercellular adhesion molecule-1 (ICAM-1) and vascular cell adhesion molecule-1 (VCAM-1) provide insights into vascular inflammation and leukocyte adhesion. While LDL-C identifies lipid-driven risk, ICAM-1 and VCAM-1 reveal early endothelial changes that predispose arteries to plaque formation. Myeloperoxidase (MPO), an enzyme involved in oxidative stress and LDL oxidation, is strongly linked to plaque vulnerability and rupture risk, offering predictive value in acute cardiovascular events, which LDL-C alone cannot provide.

Lipopolysaccharide-binding protein (LBP) is emerging as a biomarker linking gut microbiota dysbiosis to systemic inflammation. Unlike LDL-C, which focuses on lipid transport, LBP reflects metabolic endotoxemia, a novel contributor to cardiovascular disease. Elevated LBP levels indicate gut-derived inflammation, suggesting a broader systemic influence on atherosclerosis beyond traditional lipid markers.

## 3. The Role of Gut Microbiota in Atherosclerosis

The gut microbiome, consisting of trillions of microorganisms residing in the intestines, has been increasingly recognized as a critical regulator of cardiovascular health [56]. The composition of gut microbiota is highly dynamic and influenced by various factors, including diet, medication use (e.g., antibiotics), lifestyle, and genetics. Recent research suggests that gut microbial composition plays a crucial role in modulating lipid metabolism, immune system function, and systemic inflammation, all of which contribute to atherosclerosis [57,58]. Dysbiosis, or an imbalance in gut microbial populations, has been linked to heightened cardiovascular risk through various metabolic pathways that alter host metabolism and immune responses [59,60,61].

One of the most significant contributors is trimethylamine N-oxide (TMAO), a metabolite produced when gut bacteria metabolize dietary choline and carnitine, commonly found in red meat, eggs, and dairy products [62]. TMAO has been shown to enhance cholesterol deposition in arterial walls, promote platelet aggregation, and increase vascular inflammation, thereby accelerating the development of atherosclerosis [63,64]. Elevated circulating levels of TMAO have been strongly associated with increased cardiovascular risk, independent of traditional risk factors such as LDL-C levels. Studies have also suggested that TMAO may contribute to endothelial dysfunction and impair nitric oxide signaling, further exacerbating vascular damage.

Conversely, short-chain fatty acids (SCFAs), including butyrate and acetate, have demonstrated protective effects against atherosclerosis [65]. SCFAs, produced by gut bacterial fermentation of dietary fiber, exhibit potent anti-inflammatory properties and help maintain endothelial integrity by improving insulin sensitivity, reducing oxidative stress, and modulating immune responses [66,67]. A gut microbiota rich in fiber-fermenting bacteria is associated with a lower risk of cardiovascular disease, highlighting the importance of dietary interventions in modulating microbial balance [68].

Additionally, lipoprotein lipase-modulating bacteria have been identified as key regulators of lipid metabolism. Certain gut microbes influence triglyceride and LDL-C levels by altering bile acid metabolism and hepatic lipid processing. Disruptions in gut microbial balance can lead to dyslipidemia, increasing cardiovascular risk even in individuals with normal cholesterol levels [69]. Emerging evidence suggests that probiotic and prebiotic interventions can help restore microbial balance and improve lipid metabolism [70].

Modulating gut microbiota through targeted dietary interventions, such as increasing fiber intake, incorporating probiotics, and reducing red meat consumption, presents a promising avenue for reducing atherosclerosis risk [71]. Pharmacological approaches, including TMAO-lowering drugs, prebiotic supplementation, and microbial transplantation strategies, are also being explored as potential methods to mitigate the detrimental effects of dysbiosis on cardiovascular health.

Recent advancements in gut microbiome research have also pointed to the potential role of specific bacterial strains in cardiovascular protection [72]. For example, Lactobacillus and Bifidobacterium species have been shown to exert anti-inflammatory and lipid-lowering effects, while certain pathogenic strains may exacerbate systemic inflammation and metabolic dysfunction. Understanding the precise microbial signatures associated with cardiovascular risk will enable more personalized approaches to prevention and treatment [70].

**Figure 1:**
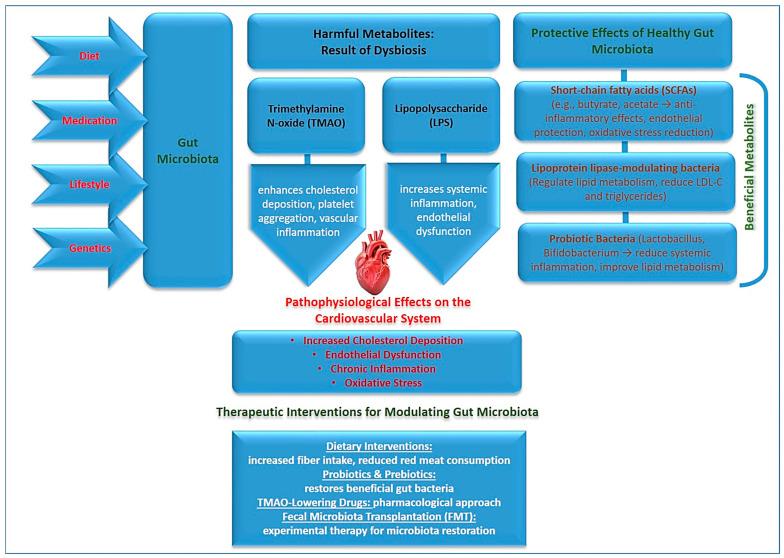
Diagram of the interactions between gut microbiota, its metabolites, and their effects on cardiovascular health.

The interaction between the gut microbiome and the immune system is another critical aspect of its role in atherosclerosis [72]. Gut-derived metabolites can influence the activity of immune cells, promoting either pro-inflammatory or anti-inflammatory responses. Increased intestinal permeability, often associated with dysbiosis, allows microbial toxins such as lipopolysaccharides (LPS) to enter systemic circulation, triggering chronic inflammation and endothelial dysfunction—both of which are key drivers of atherosclerosis progression. Figure 1 illustrates the interactions between gut microbiota, its metabolites, and their effects on cardiovascular health.

Overall, the gut microbiota represents a crucial yet underappreciated contributor to atherosclerosis. Modulating gut microbial composition through dietary, pharmacological, and lifestyle interventions offers a new and promising approach to cardiovascular disease prevention beyond traditional lipid management [71]. Further research is needed to fully elucidate the mechanisms by which gut microbes influence cardiovascular health and to develop targeted strategies for improving microbial balance and reducing cardiovascular risk. A deeper understanding of these emerging risk factors will help refine current therapeutic strategies and improve long-term cardiovascular health outcomes.

## 4. Environmental Exposures and Cardiovascular Risk

Emerging research suggests that atherosclerosis is a multifactorial disease influenced by various biological and environmental factors beyond cholesterol (Table 2). Chronic inflammation, gut microbiota composition, oxidative stress, and environmental exposures have been identified as critical contributors to disease pathogenesis. These factors interact in complex ways to influence endothelial function, immune responses, and lipid metabolism, ultimately shaping the trajectory of atherosclerosis [16,73]. Understanding these novel contributors offers a more comprehensive perspective on cardiovascular disease risk and provides opportunities for targeted interventions that extend beyond conventional lipid management.

Among these emerging factors, inflammation plays a pivotal role in atherogenesis, with inflammatory markers such as C-reactive protein (CRP), interleukin-6 (IL-6), and tumor necrosis factor-alpha (TNF-α) serving as key indicators of disease activity [22,74]. Additionally, recent discoveries in gut microbiota research have highlighted the impact of microbial metabolites like trimethylamine N-oxide (TMAO) on cardiovascular health, suggesting that dysbiosis may be a modifiable risk factor for atherosclerosis [63,75]. Oxidative stress, driven by reactive oxygen species (ROS), exacerbates endothelial dysfunction and promotes plaque instability, further contributing to disease progression. Moreover, environmental exposures, including air pollution, heavy metals, endocrine disruptors, and chronic psychological stress have been increasingly recognized as external influences that accelerate vascular inflammation and plaque formation [16,76].

Environmental exposures have emerged as significant contributors to the development and progression of atherosclerosis [16]. Increasing evidence suggests that chronic exposure to air pollutants, heavy metals, endocrine disruptors, and psychosocial stressors plays a crucial role in cardiovascular health by triggering oxidative stress, systemic inflammation, and metabolic dysregulation [77,78]. These environmental factors interact with traditional cardiovascular risk factors, exacerbating endothelial dysfunction, promoting lipid oxidation, and accelerating plaque formation.

One of the most well-documented environmental risk factors is air pollution, particularly fine particulate matter (PM2.5) and nitrogen dioxide (NO_2_) [18,79]. These airborne pollutants originate from vehicle emissions, industrial processes, and biomass combustion [80]. PM2.5 can penetrate deep into the respiratory tract and enter the bloodstream, inducing oxidative stress, systemic inflammation, and vascular dysfunction [81]. Long-term exposure to PM2.5 has been strongly linked to increased arterial stiffness, hypertension, and a higher risk of cardiovascular events such as myocardial infarction and stroke [82]. NO_2_, a gaseous pollutant primarily produced by combustion engines, has been associated with endothelial dysfunction and impaired nitric oxide signaling, further exacerbating vascular damage [83,84].

Another critical environmental factor is heavy metal exposure, including arsenic, lead, and cadmium, which have been linked to oxidative stress and vascular inflammation [85]. Arsenic, commonly found in contaminated drinking water, has been shown to promote endothelial dysfunction and disrupt mitochondrial function, leading to increased vascular injury [86]. Lead, a pervasive environmental toxin found in old paint, industrial emissions, and contaminated soil, contributes to hypertension and impaired vascular elasticity. Cadmium, often found in tobacco smoke and industrial waste, enhances inflammatory responses and interferes with lipid metabolism, further accelerating atherogenesis.

**Table 2:** Summary of environmental exposures and their impact on cardiovascular risk.

Environmental Factor	Mechanism of Action	Cardiovascular Impact	Sources/ Exposure	Mitigation Strategies	Ref.
Air Pollution (PM2.5, NO_2_, O_3_, CO, SO_2_)	Fine particulate matter (PM2.5) and gaseous pollutants induce oxidative stress, systemic inflammation, and endothelial dysfunction.	Increased risk of hypertension, myocardial infarction, stroke, atherosclerosis progression, and arterial stiffness.	Vehicle emissions, industrial pollution, biomass combustion, household cooking fuels.	Air quality regulations, urban green spaces, air purifiers, minimizing outdoor activities in high-pollution areas.	[80]
Heavy Metals (Arsenic, Lead, Cadmium, Mercury)	Promote oxidative stress, disrupt mitochondrial function, impair vascular elasticity, and interfere with lipid metabolism.	Hypertension, endothelial dysfunction, increased atherosclerosis risk, neurotoxicity.	Contaminated water, industrial emissions, tobacco smoke, lead-based paint, seafood (mercury exposure).	Water filtration systems, stringent environmental policies, safer industrial waste disposal, smoking cessation.	[85]
Endocrine-Disrupting Chemicals (BPA, Phthalates, Dioxins, PCBs)	Interfere with hormonal regulation, disrupt lipid metabolism, increase systemic inflammation, and impair insulin signaling.	Increased risk of obesity, insulin resistance, dyslipidaemia, endothelial dysfunction, and cardiovascular disease.	Plastics, food packaging, industrial solvents, personal care products, pesticides.	Using BPA-free products, reducing plastic use, stricter chemical regulations, promoting eco-friendly materials.	[87,88]
Chronic Psychological Stress (Work, Financial, Social, PTSD)	Activates the hypothalamic–pituitary–adrenal (HPA) axis, increasing cortisol levels, enhancing sympathetic nervous system activity, and promoting inflammation.	Hypertension, increased heart rate variability, metabolic syndrome, dyslipidaemia, gut microbiota disruption.	Workplace stress, financial instability, social isolation, trauma, sleep disorders.	Stress management (meditation, yoga, therapy), social support programs, workplace mental health initiatives.	[89]
Climate Change (Extreme Temperatures, Wildfires, Natural Disasters)	Heat stress, dehydration, and air pollution increase systemic inflammation and cardiovascular strain.	Higher incidence of heatstroke, dehydration-related arrhythmias, stroke, and cardiovascular events.	Global warming, increased frequency of extreme weather events, habitat destruction.	Climate adaptation policies, improved disaster preparedness, hydration strategies, cooling centers in urban areas.	[90,91]

Endocrine-disrupting chemicals (EDCs), such as bisphenol A (BPA) and phthalates, have also been implicated in cardiovascular disease [87,88]. These synthetic compounds, widely used in plastics, food packaging, and household products, interfere with hormonal regulation and metabolic homeostasis. BPA exposure has been linked to insulin resistance, obesity, and dyslipidemia, key contributors to cardiovascular risk [92]. Additionally, EDCs can promote inflammation and oxidative stress, further predisposing individuals to endothelial dysfunction and atherosclerosis [93,94].

Chronic psychological stress represents another emerging environmental risk factor that significantly impacts cardiovascular health [95]. Stress activates the hypothalamic–pituitary–adrenal (HPA) axis, leading to elevated cortisol levels, increased sympathetic nervous system activity, and heightened inflammatory responses.

Persistent psychological stress has been associated with hypertension, dyslipidemia, and increased platelet aggregation, all of which contribute to the development and progression of atherosclerosis [96]. Furthermore, chronic stress is known to disrupt gut microbiota composition, further compounding its adverse effects on cardiovascular health [89]. Measuring cortisol as a biomarker of chronic stress holds promise due to its role in regulating the hypothalamic–pituitary–adrenal (HPA) axis. Chronic stress leads to sustained cortisol elevation, contributing to hypertension, insulin resistance, endothelial dysfunction, and increased inflammation, all of which are linked to cardiovascular disease. Unlike subjective stress assessments, cortisol provides an objective biochemical measure, offering insights into HPA dysregulation and its impact on health [97]. However, its clinical utility is limited by fluctuations influenced by time of day, individual variability, and external factors like diet and sleep. Different measurement methods, including salivary, blood, urine, and hair cortisol, capture varying aspects of stress exposure but lack standardized thresholds for interpretation [98]. While useful in research settings, cortisol alone is insufficient for clinical stress assessment. A multi-biomarker approach, integrating cortisol with inflammatory markers and psychological evaluations, could enhance the detection and management of chronic stress-related health risks.

Addressing environmental exposures is crucial for mitigating cardiovascular risk. Public health initiatives aimed at improving air quality regulations, reducing industrial emissions, and promoting cleaner energy sources can help minimize exposure to harmful pollutants. Efforts to limit heavy metal contamination through stringent environmental policies, safer drinking water standards, and the reduction of lead-based products are also essential for cardiovascular disease prevention. Additionally, raising awareness about the health risks associated with endocrine disruptors and encouraging the use of safer alternatives in consumer products can contribute to reducing their impact on metabolic and cardiovascular health.

On an individual level, adopting lifestyle modifications can help reduce exposure to environmental risk factors and mitigate their effects on cardiovascular health. Strategies such as minimizing time spent in heavily polluted areas, using air purifiers, consuming a diet rich in antioxidants, and engaging in regular physical activity can help counteract the detrimental effects of environmental toxins. Stress management techniques, including mindfulness meditation, yoga, and cognitive behavioral therapy, have also been shown to lower cortisol levels and improve cardiovascular outcomes.

Future studies should focus on elucidating the molecular mechanisms underlying the interactions between environmental exposures and cardiovascular disease. Developing targeted interventions, including pharmacological agents that mitigate oxidative stress and inflammation induced by pollutants, may offer new therapeutic avenues for reducing the global burden of atherosclerosis. By integrating environmental risk factors into cardiovascular disease prevention strategies, we can create a more comprehensive approach to reducing cardiovascular morbidity and mortality worldwide.

## 5. The Role of Imaging in Atherosclerosis: Coronary and Vascular Calcifications

Advanced imaging techniques have become an essential tool in the diagnosis, monitoring, and risk stratification of atherosclerosis, offering insights beyond traditional lipid assessments. While elevated LDL cholesterol remains a well-established risk factor for atherogenesis, a significant proportion of cardiovascular events occur in individuals with normal or well-managed lipid profiles. This paradox highlights the need for alternative markers that can more accurately predict cardiovascular risk and disease progression. Among these, coronary and vascular calcifications have emerged as highly reliable indicators of subclinical and advanced atherosclerosis, providing direct anatomical evidence of arterial damage and plaque burden.

Coronary artery calcium (CAC) scoring, performed through non-contrast computed tomography, quantifies the extent of calcified atherosclerotic plaques within the coronary arteries [99]. Unlike lipid-based biomarkers, CAC directly measures disease presence, offering superior predictive value for future cardiovascular events such as myocardial infarction and stroke [100]. The Agatston Score, commonly used to quantify CAC, stratifies patients into risk categories, guiding the intensity of preventive interventions. Individuals with a CAC score of zero have an extremely low 10-year risk of cardiovascular events, making them less likely to require aggressive pharmacological interventions. Conversely, those with moderate-to-high CAC scores are at substantially greater risk, warranting intensive lipid-lowering therapies, lifestyle modifications, and potentially novel anti-inflammatory treatments [99,101]. Studies have consistently demonstrated that CAC is a stronger predictor of cardiovascular risk than LDL-C alone, reinforcing the need to integrate it into routine clinical practice for more accurate risk assessment [100].

Beyond the coronary arteries, vascular calcifications in the aorta and carotid arteries provide additional insights into systemic atherosclerosis. Aortic calcification, detectable through chest X-rays, CT scans, or abdominal ultrasound, is strongly associated with hypertension, arterial stiffness, and increased cardiovascular mortality [102,103]. Its presence often indicates long-standing vascular damage and is particularly prevalent among older individuals, those with metabolic disorders, and patients with chronic kidney disease. Carotid artery calcifications, visualized through ultrasound or CT angiography, are equally significant, as they correlate with an increased risk of ischemic stroke and cognitive decline [104,105]. The presence of calcified carotid plaques suggests an elevated burden of non-calcified, rupture-prone plaques, which are more likely to cause cerebrovascular events. Additionally, carotid intima-media thickness (CIMT), an ultrasound-based measurement, is frequently used to assess subclinical atherosclerosis, particularly in younger or intermediate-risk individuals, offering a non-invasive method for early disease detection.

Peripheral and intracranial artery calcifications further highlight the systemic nature of atherosclerosis, extending its implications beyond the heart and major arteries. Peripheral artery calcifications, commonly detected in the femoral and popliteal arteries via Doppler ultrasound or CT angiography, are closely linked to peripheral artery disease (PAD), which itself is associated with increased cardiovascular morbidity and mortality [106,107]. Similarly, intracranial artery calcifications, frequently identified in brain CT scans, have been correlated with a heightened risk of stroke and vascular dementia. The identification of such calcifications is critical for refining cardiovascular risk stratification, as they provide direct evidence of widespread arterial involvement and help clinicians anticipate complications before clinical symptoms arise.

The integration of vascular imaging into routine cardiovascular risk assessments represents a significant advancement in personalized medicine, enabling more precise risk prediction and tailored intervention strategies. While traditional lipid profiles, including LDL-C and lipoprotein(a) [Lp(a)], remain essential in cardiovascular risk evaluation, imaging provides direct, tangible evidence of disease burden, allowing for a more comprehensive assessment. CAC scoring, for instance, has proven particularly valuable in guiding statin therapy decisions for intermediate-risk individuals, ensuring that treatment is appropriately directed toward those who will benefit most [108,109]. This approach prevents unnecessary medication use in low-risk individuals while reinforcing the need for intensive therapy in those at high risk.

The future of atherosclerosis imaging is rapidly evolving with the incorporation of artificial intelligence (AI) and machine learning algorithms that enhance automated plaque detection, calcification quantification, and risk prediction [110]. AI-driven imaging analytics can rapidly assess large datasets, improve diagnostic accuracy, and identify subtle patterns that may be overlooked by traditional methods [111,112]. Emerging techniques, including positron emission tomography (PET) imaging for vascular inflammation and molecular imaging of calcification activity, hold promise for even earlier detection of atherosclerosis and monitoring of disease progression at a cellular level. These advancements could revolutionize the way cardiovascular disease is diagnosed and managed, moving beyond conventional cholesterol-centric models to a more precise, individualized risk assessment framework.

As cardiovascular disease remains the leading cause of mortality worldwide, combining vascular imaging with emerging biomarkers such as inflammatory markers and gut microbiota-derived metabolites will enable more effective early detection, prevention, and treatment strategies. Given the persistent cardiovascular risk that remains despite lipid-lowering therapies, adopting a multimodal approach that integrates lipid profiling with advanced imaging will provide clinicians with a more accurate and predictive risk assessment tool, ultimately improving patient outcomes.

## 6. Future Directions

Inflammatory biomarkers hold significant promise for advancing cardiovascular risk prediction, improving disease monitoring, and guiding personalized therapies in atherosclerosis. As research continues to uncover their mechanistic roles in plaque progression and instability, future applications of these biomarkers could revolutionize cardiovascular medicine by shifting the focus from traditional lipid-based assessments to inflammation-driven precision medicine.

One of the most promising applications of inflammatory biomarkers lies in the enhancement of cardiovascular risk stratification. Current risk models primarily rely on lipid levels, blood pressure, and lifestyle factors, yet these fail to fully account for the role of chronic inflammation in atherosclerosis. Incorporating biomarkers such as high-sensitivity C-reactive protein (hs-CRP), interleukin-6 (IL-6), tumor necrosis factor-alpha (TNF-α), and monocyte chemoattractant protein-1 (MCP-1) into predictive models could help identify high-risk individuals who may not have elevated low-density lipoprotein cholesterol (LDL-C) but still harbor significant atherosclerotic disease. Future risk calculators may use multi-marker panels that combine inflammatory and lipid biomarkers to provide more accurate, individualized assessments of cardiovascular risk. This approach could enable earlier intervention and more effective allocation of preventive therapies to individuals who would benefit most from aggressive risk reduction strategies.

Another major advancement is the potential for personalized anti-inflammatory therapies tailored to individual biomarker profiles. The CANTOS, which demonstrated that IL-1β inhibition significantly reduces cardiovascular events, highlights the potential for targeting inflammatory pathways in atherosclerosis treatment. Future applications may include precision medicine approaches where patients with elevated IL-6 or IL-1β levels receive targeted anti-inflammatory therapies such as canakinumab or colchicine. Additionally, leukotriene inhibitors, such as zileuton and montelukast, could be explored as adjunct therapies for patients with elevated leukotriene B4 (LTB4) levels, particularly those with co-existing inflammatory conditions such as asthma or autoimmune disorders. Combination therapies integrating lipid-lowering agents like statins or PCSK9 inhibitors with anti-inflammatory drugs may offer a dual-action approach to cardiovascular risk reduction, addressing both hyperlipidemia and chronic vascular inflammation.

Non-invasive monitoring of atherosclerotic activity using inflammatory biomarkers represents another exciting frontier. Biomarkers such as TNF-α, IL-6, and leukotrienes could be used for real-time tracking of atherosclerosis progression, allowing for earlier intervention before clinical symptoms arise. Liquid biopsy techniques that analyze inflammatory markers from blood samples could reduce reliance on imaging-based assessments, providing a less invasive and more cost-effective means of monitoring disease activity. Emerging wearable or implantable biosensors may soon allow for continuous tracking of inflammatory biomarkers, enabling real-time risk assessment and early detection of plaque instability or impending cardiovascular events. This could lead to a new era of precision monitoring, where high-risk individuals can be proactively managed based on dynamic biomarker changes rather than waiting for clinical manifestations of disease.

Targeted prevention strategies based on inflammatory biomarker profiling could also play a significant role in reducing the burden of cardiovascular disease. Early detection of subclinical inflammation in individuals with metabolic syndrome or a genetic predisposition to atherosclerosis could enable proactive lifestyle and pharmacological interventions. Patients with elevated CRP and IL-6 levels but normal lipid profiles could be recommended intensive lifestyle interventions, including the Mediterranean diet, structured exercise programs, and anti-inflammatory supplements such as polyphenols and omega-3 fatty acids. Personalized exercise programs tailored to lower inflammation in at-risk individuals could be designed based on biomarker profiling, optimizing cardiovascular benefits while minimizing systemic inflammation.

Another emerging area of interest is the integration of inflammatory biomarkers into artificial intelligence (AI)-driven predictive models. AI and machine learning have the potential to revolutionize how inflammatory biomarkers are used in clinical decision making by analyzing large multi-biomarker datasets to predict which patients are most likely to develop cardiovascular events. Deep learning models may identify novel biomarker interactions that are currently underappreciated in standard risk models, leading to new insights into disease mechanisms and risk prediction. AI-assisted precision medicine may help determine the most effective anti-inflammatory therapies for individual patients based on their biomarker profiles, optimizing treatment efficacy while minimizing unnecessary interventions.

As the field of cardiovascular medicine continues to evolve, the role of inflammatory biomarkers is expected to expand, providing more precise risk assessment tools, better treatment personalization, and improved monitoring strategies. By integrating inflammatory biomarker panels, precision therapies, non-invasive monitoring, and AI-driven predictive tools, clinicians will be better equipped to identify high-risk individuals, personalize treatment plans, and ultimately reduce the global burden of atherosclerosis-related morbidity and mortality. The transition from traditional lipid-centric models to inflammation-targeted strategies represents a paradigm shift that could lead to more effective prevention and management of cardiovascular disease in the future.

## 7. Conclusions

Atherosclerosis is a complex, multifactorial disease influenced by more than just cholesterol levels. Emerging evidence highlights the significant contributions of inflammation, gut microbiota, oxidative stress, and environmental exposures to its pathogenesis. Inflammation is a key driver of atherosclerosis, with CRP, IL-6, and TNF-α serving as markers of disease activity. The gut microbiome plays a crucial role in modulating lipid metabolism and immune responses, with metabolites like TMAO exacerbating cardiovascular risk while SCFAs provide protective effects. Oxidative stress disrupts vascular homeostasis, promoting endothelial dysfunction, lipid oxidation, and plaque instability. Additionally, environmental factors such as air pollution, heavy metals, endocrine disruptors, and chronic stress contribute to systemic inflammation and vascular damage. Addressing these emerging risk factors requires a comprehensive, multi-faceted approach integrating public health initiatives, lifestyle modifications, and novel therapeutic strategies. Targeted interventions, including anti-inflammatory treatments, microbiome-based therapies, antioxidant support, and pollution mitigation efforts, may enhance traditional lipid-lowering therapies. Future research should focus on incorporating these factors into cardiovascular risk models to improve early detection, treatment personalization, and overall disease management. Expanding our understanding of atherosclerosis beyond cholesterol will enable more effective strategies to reduce its prevalence and associated complications, ultimately improving global cardiovascular health outcomes.
